# The Prevalence of Depression in Survivors of Acute Myocardial Infarction and Gender Differences in King Abdulaziz Medical City (KAMC), Riyadh

**DOI:** 10.7759/cureus.57456

**Published:** 2024-04-02

**Authors:** Ihab Suliman, Hanan A Almkainzi, Abdullah M Alsubaie, Faisal N Alqahtani, Faris A Alkhudairy, Osama Alrodiman, Alwaleed K Nahhas, Abdulaziz M Alnasser

**Affiliations:** 1 Cardiology, King Abdulaziz Medical City, King Abdulaziz Cardiac Center, Ministry of National Guard Health Affairs, Riyadh, SAU; 2 Medicine, King Saud bin Abdulaziz University for Health Sciences (KSAU-HS), Riyadh, SAU; 3 Internal Medicine, King Saud bin Abdulaziz University for Health Sciences (KSAU-HS), Riyadh, SAU; 4 Psychiatry, King Abdulaziz Medical City, Riyadh, SAU; 5 Otolaryngology, King Abdulaziz Medical City, Riyadh, SAU; 6 Internal Medicine, Security Forces Hospital, Riyadh, SAU; 7 Psychiatry Department, Prince Sultan Military Medical City, Riyadh, SAU

**Keywords:** post-mi, cardiology, mental health, myocardial infarction, depression

## Abstract

Background: Myocardial infarction (MI) stands as a prevalent worldwide cause of mortality. The aftermath of an MI often entails an unpleasant experience for individuals, who frequently find themselves overwhelmed. Extensive literature suggests that a significant proportion of patients develop depressive symptoms subsequent to MI. Consequently, the primary objective of this study is to ascertain the prevalence of post-MI depression among affected individuals.

Methods: This is a cross-sectional study involving a survey distributed to patients admitted to the King Abdulaziz Medical Center (KAMC) located in Riyadh, Saudi Arabia. The study involved 210 patients; 72.1% of the sample were men and 27.9% were female.

Results: The average age of the participants in this study was 61.96 years old. The mean age of the male participants was 61.10 years old, while the females’ mean age was 64.35 years old. Males made up 75.27% of the participants, while 24.73% were females. Overall, 33.64% of the participants had an abnormal score (depression). The majority of male patients had a normal score, which means that they do not suffer from depression. Among the female patients, 38.60% had an abnormal score.

Conclusion: Mental illness is a significant concern, particularly depression. Individuals should go for depression screening post-MI as it will determine their compliance with visiting the hospital, caring for themselves, and taking medications.

## Introduction

Myocardial infarction (MI) is the most common cause of death globally. It is defined as irreversible damage to the heart muscles caused by a lack of blood supply. One of the most common ways MI can develop is through the presence of atherosclerotic plaques that promote clot formation and therefore cut the supply of blood to the heart muscles. MI was estimated to affect three million people around the world. In addition, MI is responsible for other heart diseases, such as heart failure, left ventricular dysfunction, arrhythmias, and sudden cardiac death [1]. Multiple factors increase the likelihood of an MI. Some are non-modifiable, such as age, family history, and male gender. Others are modifiable factors, such as uncontrolled blood pressure, uncontrolled cholesterol levels, and smoking [2]. MI is also a very prevalent problem in our population. In a study conducted in Saudi Arabia in 2004, the overall prevalence of coronary artery disease was reported to be 5.5%; it was higher in males (6.6%) than in females (4.4%) [3]. Other studies have shown that the Saudi population is genetically more susceptible to coronary artery disease [4].

There is a correlation between the occurrence of an MI and depression. According to multiple meta-analysis studies, depression following an MI is highly prevalent, affecting between 18.6% and 28.7% of patients [5,6]. One meta-analysis including studies from 10 different countries showed that the rate of post-MI depression varied significantly between countries, ranging from 9.17% to 65.88%, with a pooled prevalence of 28.7%. A 2004 study conducted in the eastern region of Saudi Arabia found depression to affect 20.6% of patients post-MI [7]. Another local study, which examined patients in Al-Qassim who had an MI between 2008 and 2015, concluded that 25% of patients had depression post-MI [8]. It has also been found that women have a notably higher prevalence of depression post-MI compared to men, with 36% in women and 29% in men. However, the all-cause mortality associated with depression is higher in men, and men with depression post-MI have a worse prognosis than women with depression post-MI [9,10].

Patients with depression are less likely to practice self-care and follow medical advice, and it was documented that heart failure patients with depression were less likely to stop smoking, exercise, or adhere to medical treatment. They are also less likely to seek medical attention when they have symptoms [11]. Moreover, depression lowers the quality of life and contributes to the deterioration of health post-MI. According to a meta-analysis studying the prognosis of patients who have depression post-MI, the prevalence of all-cause mortality and cardiovascular events was found to be higher among the depressed patient group versus the non-depressed patient group [12]. Therefore, it is crucial to recognize patients with depression early, as the resolution of depression improves the cardiovascular prognosis. Recognizing the problem early enables multiple management options, such as psychotherapy, pharmacotherapy, and exercise [13].

Two questionnaires are commonly used in studies on post-MI depression: Beck's Depression Inventory (BDI) and the Hospital Anxiety and Depression Scale (HADS) (see Appendix) [14]. In the current study, an Arabic version of HADS was used to assess the prevalence of depression. The HADS scale was validated by Terkawi [15].

## Materials and methods

Study settings and sample size

This observational cross-sectional study was conducted in King Abdulaziz Medical City/King Abdulaziz Cardiac Center in Riyadh, Saudi Arabia. The study included all patients who had MIs in 2022. Information about the patients was gathered using an Arabic version of the HADS that has been validated by Terkawi [16]. The data were collected using a convenient data collection form, and each patient was interviewed individually by the researchers to ensure confidentiality. Data analysis was done using IBM SPSS Statistics (IBM Corp., Armonk, NY).

Approval for the study was obtained from King Abdullah International Medical Research Center (KAIMRC), and consent was obtained from patients before they were interviewed.

Study measures

The study gathered demographic data, such as gender and age. In addition, the HADS was used to measure the levels of anxiety and depression in the hospital setting. The scale consists of 14 items: seven items concerning depression and seven items to assess anxiety. Each item is scored on a scale of 0 to 3, giving a maximum score of 21 for each of the two subscales. The interpretation of the results is as follows: 0-7 is considered a normal range; 8-10 is suggestive of the presence of a state of either anxiety or depression, depending on the subscale; and a score of 11 or higher indicates the presence of a mood disorder.

Data analysis

The data were analyzed using IBM SPSS Statistics for Windows, version 26.0 (released 2019, IBM Corp., Armonk, NY). Descriptive statistics (frequencies and percentages) were used to describe the categorical study and outcome variables. Pearson’s chi-square test was used to observe the association between the categorical variables and compare the distribution of proportions across the two categorical variables.

## Results

Among 214 MI cases, we observed 33.18% normal, 33.18% borderline abnormal, and 33.6% abnormal. Among the male participants, 35.03% were normal, 33.12% were borderline abnormal, and 31.85% were abnormal. Of the female cases, 28.07% were normal, 33.33 % were borderline abnormal, and 38.60 % were abnormal (Table 1). 

**Table 1:** Types of depression

Variable	Sub-variable	Frequency	Percentage (%)
Depression			
	Normal	71	33.18
	Borderline abnormal	71	33.18
	Abnormal	72	33.64
Depression (male)			
	Normal	55	35.03
	Borderline abnormal	52	33.12
	Abnormal	50	31.85
Depression (female)			
	Normal	16	28.07
	Borderline abnormal	19	33.33
	Abnormal	22	38.60

The overall mean age was 61.90 ± 14.74 years. The average age of the males and females was 61.10 ± 14.01 and 64.35 ± 16.52, respectively. Among the 279 patients observed in our study, 24.73% were females and 75.27% were males (Table 2).

**Table 2:** Demographic variables

Variable	Sub-variable	Mean/frequency	Standard deviation/percentage
Age		61.90	14.74
Age (male)		61.10	14.01
Age (female)		64.35	16.52
Gender			
	Female	69	24.73
	Male	210	75.27

The chi-square test was used to find out the association between gender and type of depression (normal, borderline abnormal, and abnormal). Out of 214 cases, we obtained 22 abnormal scores among females and 50 abnormal scores among males. We did not observe a significant association between gender and the type of depression (Chi-square: 1.178, p = 0.5550; Table 3).

**Table 3:** Association between type of depression and gender

		Type of depression				
		Normal	Borderline abnormal	Abnormal	Total	p-value
Gender	Female	16	19	22	57	0.5550
	Male	55	52	50	157	
	Total	71	71	72	214	

## Discussion

The goal of this study was to investigate how common depression is among patients post-MI. The average age of the study participants was approximately 61.90 years; the male participants averaged 61.10 years, and the females had an average age of 64.35 years. Of the 210 study participants, 75.27% were men (n = 210) and roughly 24.73% were women (n = 69). About 33.64% of the patients experienced depression, indicated by an abnormal score. The male participants exhibited scores within the standard range, while the female participants displayed scores that deviated significantly, indicating the presence of depression. Kjellstrom and Gustafsson found that 39% of the patients who had an MI suffered from depression [16]. Zheng et al. showed that the percentage of people who had depression post-MI was 50% [17]. Our finding that 33.64% of patients post-MI had abnormal scores, 33.18% had borderline scores, and 33.18% had normal scores supports previous studies, indicating that most people with post-MI are at risk of depression.

Our findings showed that female patients have a higher likelihood of depression than male patients. The majority of female patients had an abnormal score (38.60%), which was higher than the percentage of males with a depression score (31.85%). Shanmugasegaram et al. found that the risk of depression among females with cardiac heart disease is 1.77 times higher than that of males [18]. Moreover, AbuRuz et al. and Wang et al.'s studies further support our finding that females are at a higher risk of developing depression post-MI compared to male patients [19,20]. Frasure-Smith et al.’s study used BDI on all patients and indicated that female patients have a high prevalence of depression [21]. 

Our study supports previously published literature, showing that individuals who have had an MI might experience stress and should get a depression screening. If patients are screened, it will be much easier for them to adhere to their prescriptions and therapies as prescribed. Possible future explorations into this research question should begin by increasing the sample size to assess more of the population and get more accurate calculations.

The limitations of our paper are the sample size and participation of MI patients as interviews were done in a tight-timed clinic setting. Furthermore, the difference between the number of male and female participants is apparent. Possible future expansions regarding answering this research question should be by increasing the sample size to both assess more of the population and to get more accurate calculations.

## Conclusions

The findings of our study revealed a notably elevated occurrence of depression among individuals diagnosed with acute myocardial infarction. Among these patients, females exhibited a greater susceptibility to developing depression following an acute MI compared to males. In addition, our analysis did not establish any significant correlation between age and depression, nor did it find a significant association between the type of depression and the occurrence of MI in either gender. Recognizing and promptly addressing depression in patients is of utmost importance, as it enables effective management and enhances overall quality of life.

## Figures and Tables

**Figure 1:**
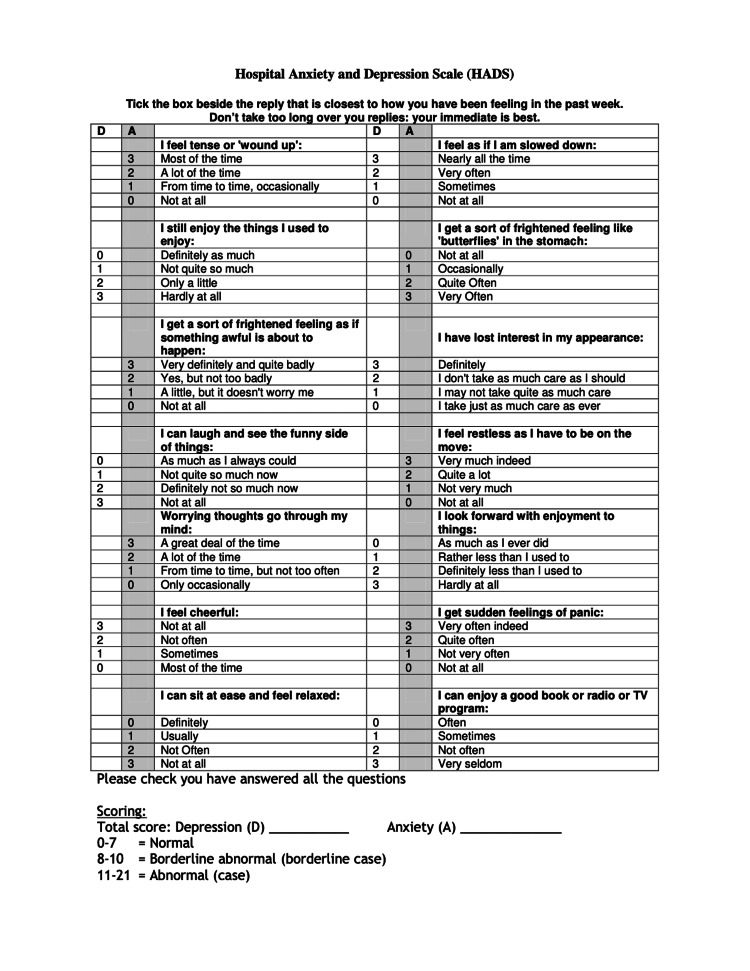
Hospital Anxiety and Depression Scale (HADS)

## References

[1] Mechanic O (2023). Acute myocardial infarction. StatPearls [Internet].

[2] (2011). Million hearts: strategies to reduce the prevalence of leading cardiovascular disease risk factors--United States, 2011. MMWR Morb Mortal Wkly Rep.

[3] Al-Nozha MM, Arafah MR, Al-Mazrou YY (2004). Coronary artery disease in Saudi Arabia. Saudi Med J.

[4] Wakil SM, Ram R, Muiya NP (2016). A genome-wide association study reveals susceptibility loci for myocardial infarction/coronary artery disease in Saudi Arabs. Atherosclerosis.

[5] Thombs BD, Bass EB, Ford DE (2006). Prevalence of depression in survivors of acute myocardial infarction: review of the evidence. J Gen Intern Med.

[6] Larsen KK (2013). Depression following myocardial infarction--an overseen complication with prognostic importance. Dan Med J.

[7] Feng L, Li L, Liu W, Yang J, Wang Q, Shi L, Luo M (2019). Prevalence of depression in myocardial infarction: a PRISMA-compliant meta-analysis. Medicine (Baltimore).

[8] Doyle F, McGee H, Conroy R (2015). Systematic review and individual patient data meta-analysis of sex differences in depression and prognosis in persons with myocardial infarction: a MINDMAPS study. Psychosom Med.

[9] Saquib J, AlRomaih NA, Al-Mutairi HM (2018). Correlates of memory loss and depression among myocardial infarction patients in Al-Qassim, Saudi Arabia. J Saudi Heart Assoc.

[10] Meijer A, Conradi HJ, Bos EH (2013). Adjusted prognostic association of depression following myocardial infarction with mortality and cardiovascular events: individual patient data meta-analysis. Br J Psychiatry.

[11] Riegel B, Lee CS, Dickson VV (2011). Self care in patients with chronic heart failure. Nat Rev Cardiol.

[12] Abdul-Mohsen MF (2004). Frequency of depression among patients with acute coronary syndrome, eastern region, saudi arabia. J Family Community Med.

[13] Liblik K, Mulvagh SL, Hindmarch CC, Alavi N, Johri AM (2022). Depression and anxiety following acute myocardial infarction in women. Trends Cardiovasc Med.

[14] Shapiro PA (2015). Management of depression after myocardial infarction. Curr Cardiol Rep.

[15] Terkawi AS, Tsang S, AlKahtani GJ (2017). Development and validation of Arabic version of the Hospital Anxiety and Depression Scale. Saudi J Anaesth.

[16] Kjellström B, Gustafsson A, Nordendal E (2017). Symptoms of depression and their relation to myocardial infarction and periodontitis. Eur J Cardiovasc Nurs.

[17] Zheng X, Zheng Y, Ma J (2019). Effect of exercise-based cardiac rehabilitation on anxiety and depression in patients with myocardial infarction: A systematic review and meta-analysis. Heart Lung.

[18] Shanmugasegaram S, Russell KL, Kovacs AH, Stewart DE, Grace SL (2012). Gender and sex differences in prevalence of major depression in coronary artery disease patients: a meta-analysis. Maturitas.

[19] AbuRuz ME, Alaloul F, Al-Dweik G (2018). Depressive symptoms are associated with in-hospital complications following acute myocardial infarction. Appl Nurs Res.

[20] Wang W, Thompson DR, Ski CF, Liu M (2014). Health-related quality of life and its associated factors in Chinese myocardial infarction patients. Eur J Prev Cardiol.

[21] Frasure-Smith N, Lespérance F, Talajic M (1993). Depression following myocardial infarction. Impact on 6-month survival. JAMA.

